# Core–shell silica–rhodamine B nanosphere for synthetic opals: from fluorescence spectral redistribution to sensing†

**DOI:** 10.1039/d0ra02245d

**Published:** 2020-04-16

**Authors:** Paola Lova, Simone Congiu, Katia Sparnacci, Angelo Angelini, Luca Boarino, Michele Laus, Francesco Di Stasio, Davide Comoretto

**Affiliations:** Dipartimento di Chimica e Chimica Industriale, Università degli Studi di Genova Via Dodecaneso 31 16132 Genova Italy paola.lova@edu.unige.it; Dipartimento di Scienze e Innovazione Tecnologica (DISIT), Università del Piemonte Orientale “A. Avogadro”, INSTM, UdR Alessandria Viale T. Michel 11 15121 Alessandria Italy katia.sparnacci@uniupo.it; Quantum Research Labs & Nanofacility Piemonte, Advanced Materials Metrology and Life Science Division, Istituto Nazionale di Ricerca Metrologica (INRiM) Strada delle Cacce 91 Torino IT10135 Italy

## Abstract

Photonic crystals are a unique tool to modify the photoluminescence of light-emitting materials. A variety of optical effects have been demonstrated by infiltrating opaline structures with photoactive media. On the other hand, the fabrication of such structures includes complex infiltration steps, that often affect the opal lattice and decrease the efficiency of light emission control. In this work, silica nanospheres were directly functionalized with rhodamine B to create an emitting shell around the dielectric core. Simple tuning of the microsphere preparation conditions allows selecting the appropriate sphere diameter and polydispersity index approaching 5%. These characteristics allow facile self-assembling of the nanospheres into three-dimensional photonic crystals whose peculiar density of photonic states at the band-gap edges induces spectral redistribution of the rhodamine B photoluminescence. The possibility to employ the new stable structure as sensor is also investigated. As a proof of principle, we report the variation of light emission obtained by exposure of the opal to vapor of chlorobenzene.

## Abstract 2

Silica nanospheres were functionalized with rhodamine B and self-assembled into opals. Photoluminescence redistribution and pollutant responsivity were also investigated.

## Graphical abstract

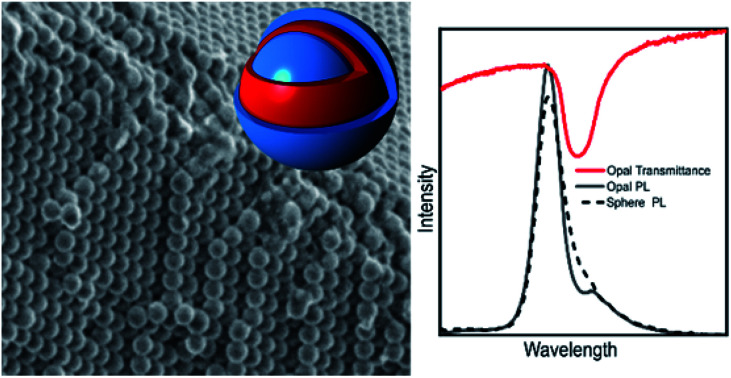

## Introduction

Monodisperse spherical colloids with submicrometric diameter are widely researched for several photonic applications including structural color, lithography templates,^1–3^ plasmonic structures,^4–6^ chemical sensing,^7–11^ pollution treatment,^12,13^ water splitting,^14–17^ and the performance enhancement of polymer and hybrid photovoltaic^18–20^ and light emitting devices.^21^ Colloidal nanospheres are indeed often used as building-blocks for self-assembled highly ordered two- or three-dimensional photonic systems called monolayers^5,22^ and opals.^23–26^ Different from other photonic crystals, widely researched in sensing^27–33^ and light emission control,^19,28,34^ opals are an easy tool to tailor photophysical properties and light–matter interaction by solution processing,^10,35–41^ also on large area.^42^ Highly ordered lattices can be obtained using nearly monodispersed nanospheres with the possibility to impart optical properties *ad hoc* by simple material engineering depending on the required application. As an example, photoemitters can be coupled with the opaline lattice by infiltration of dye solutions in the porosity of the structure. At times, dissolution of the opaline template can be performed, leading to inverse opals. On the other hand, infiltration may negatively affect the order and the periodicity of the lattice, thus its optical response. Embedding the photoemitter within the lattice during the synthesis of the colloidal system would avoid the formation of defects occurring during the infiltration. This approach has been demonstrated highly effective in the preparation of polymer nanospheres with dyes containing a polymerizable moiety. They dyes are incorporated in the nanospheres during emulsifier-free or seeded emulsion polymerization, whereas non-polymerizable dyes can be included into preformed nanoparticles by a swelling and de-swelling process during the synthetic procedure.^43,44^ Furthermore, colloids with core–shell structure containing photo-active molecules or noble metal nanoparticles were also synthesized employing charged nanosphere surfaces.^43^

In this work, we demonstrate a similar approach to fabricate self-assembled opals made of fluorescent silica nanospheres. These nanospheres were obtained through a multistep procedure that allowed to incorporate the fluorophore, rhodamine B (RhDB), in a thin layer inside the particle matrix. Similar synthetic methods can be used for functionalization of the silica spheres with commercial dyes^45^ and metal chelates providing characteristic sharp emission^46^ that makes the microspheres interesting for cell imaging. The possibility to include also quantum-dots in the silica matrix makes the structure appealing for lightening devices^47,48^ and lasing action.^49^ Near-infrared emitters can instead be used for applications in the telecommunication spectral range.^50^ Moreover, the specific synthetic procedure adopted in this work, can be easily extended, besides RhDB, to all amine-reactive fluorophores, allowing to obtain fluorescent particles with emission in the entire visible and near-infrared spectrum.^51^

The diameter of the colloidal spheres, together with the dye concentration, are engineered to favour the spectral superimposition between the RhDB photoluminescence and the stop-band of the self-assembled opals. The performances of the structure are quantified by analysing the modifications occurring on the RhDB photoemission in term of intensity and lines-shape. This approach allows a stable and robust solid-state photonic structure to be obtained, potentially useful in several applications. As an example of a future application, we report as a proof-of-principle, the optical response of the opaline structure to the exposure of chlorobenzene vapors.

## Experimental section

### Nanosphere synthesis

Fluorescent silica nanospheres were obtained through a multistep procedure illustrated in Fig. S1:† first, monodispersed silica nanospheres were synthesized by means of a modified Stöber method^52^ which involve hydrolysis and condensation of tetraethylorthosilicate (TEOS) in a water–ethanol mixture with ammonia as a basic catalyst. By appropriately adjusting the ammonia concentration it is possible to control the obtained particle size^53^ and in this case a nanoparticle sample with diameter of 210 nm was prepared. Then, a fluorescent silica shell 15 nm thick was growth on these particles using a functional fluorescent dye, obtained by reacting rhodamine B isothiocyanate with 3-aminopropyl triethoxysilane (Fig. S2†). Finally, the obtained fluorescent particles were covered by a 10 nm thick silica layer, to protect the fluorophore from hydrolysis (synthetic details are reported in ESI†).

### Photonic crystal fabrication

The nanospheres suspension was diluted in de-ionized water as necessary to obtain the desired film thickness upon complete water evaporation inside a BF53 Binder incubator. The growth process was carried out at 40 °C ± 1 °C on soda-lime glass slides using the meniscus technique which yields a face-centered cubic lattice of nanospheres with the [111] direction perpendicular to the substrate over a 5 × 5 mm area (the evaporation process required around 60 h).

### Photonic crystal characterization

Reflectance spectra were measured using a system based on an Avantes spectrometer (resolution 1.4 nm) as previously described. Angle resolved spectra have been recorded by a homemade setup (angular resolution ≤ 1°). Steady-state PL measurements were performed by exciting the samples with an Oxxius 405 nm CW laser focused on a 1 mm^2^ spot and the fluorescence was collected with the same Avantes compact spectrometer. Time-resolved PL measurements were recorded using a 405 nm LDH-P-C-405 laser combined with a PDL 800B driver (40 ps pulse width, 5 MHz repetition rate) and a PicoQuant time correlated single photon counting system (Time Harp 260 PICO board, 25 ps temporal resolution; PMA Hybrid 40 detector, 140 ps response time) equipped with a Solar Laser Systems monochromator.

### Photonic crystal sensor test

The sensor response was measured by a fibre-based set-up consisting of a cuvette holder with SMA fibers connectors. The sample contained in the cuvette is excited by the 405 nm CW laser previously described and photoemission is collected at 90° with a fibre connected to an Avantes 2048 CCD photodetector with set time interval. The sample chamber was connected to a liquid reservoir allowing analyte vapors to reach the sample as schematized in Fig. 5a. The measurements were performed at 22 °C and 1 atm in room conditions.

SEM micrographs were collected using Field Emission Gun Inspect F microscope from FEI Company, with a nominal beam diameter of 3 nm. The SEM micrographs were elaborated by the NIH ImageJ, a Java version of the former NIH Image software. From 200 to 250 individual particles were measured for each sample.

## Results and discussion

**Fig. 1:**
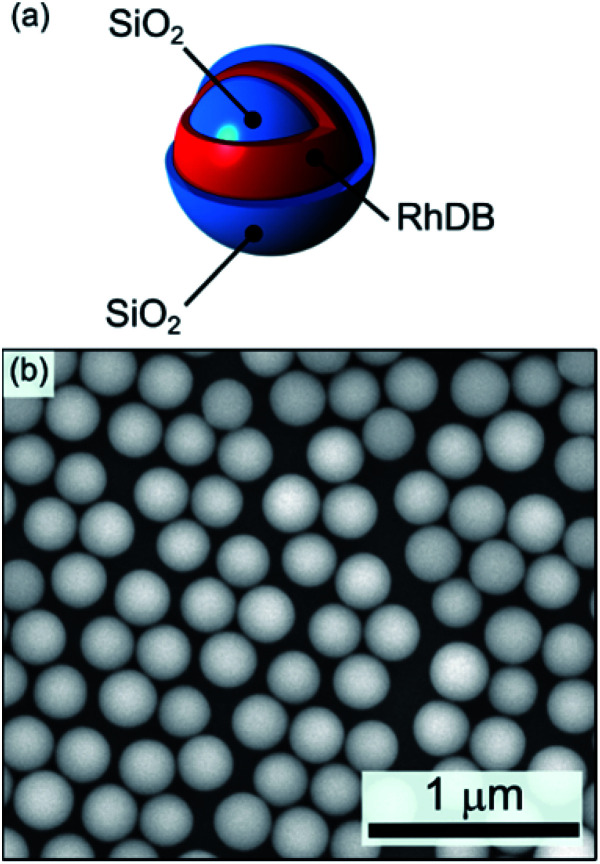
(a) Schematic and (b) SEM micrograph of the fluorescent silica nanospheres (average diameter 277 ± 13 nm).

Through a multistep procedure based on a modified Stöber method we prepared a fluorescent silica nanoparticle sample with RhDB incorporated in a thin layer inside the particle matrix, as sketched in Fig. 1a. Fig. 1b reports a SEM micrograph of the final colloidal particles. The particles are highly monodispersed with a final diameter of 261 ± 15 nm. Under laser excitation, these nanospheres show a photoluminescence (PL) spectrum peaked at 588 nm (ESI Fig. S3†). The signal arises from the RhDB layer enclosed in the nanosphere surface and is consistent with the emission of the dye.^54,55^ The opaline structures were self-assembled as described in the Experimental section, by diluting the nanosphere colloidal solutions in water to concentrations of approximately 21.5 mg ml^−1^.^23,56^

**Fig. 2:**
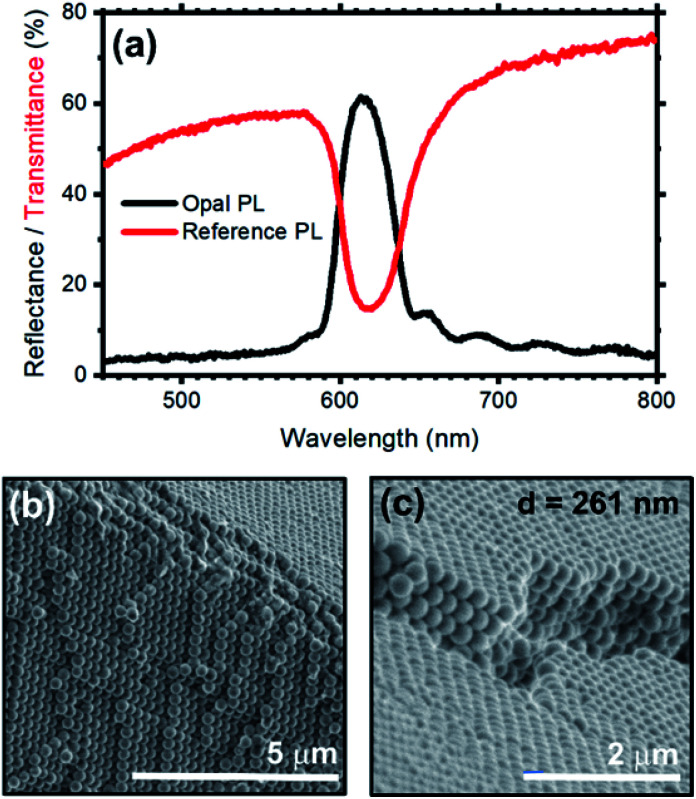
(a) Reflectance (black curve) and transmittance (red curve) spectra of an opal fabricated *via* vertical-deposition on a soda-lime glass slide employing the fluorescent silica nanospheres. As expected, the optical features (*i.e.* reflectance peak and transmission minimum) of the photonic band gap overlap, and they are centered at 616 nm. Interference fringes are visible in the reflectance spectrum indicating the high optical quality of the obtained sample. SEM micrographs of the opal: (b) cross-section and (c) surface. In both micrographs the face-centered-cubic lattice of the nanospheres can be appreciated.

Fig. 2a shows the transmittance (red line) and reflectance (black line) spectra collected for a self-assembled opal fabricated. The bottom panels (Fig. 2b and c) show instead the SEM characterization of the resulting opal.

Regarding the optical characterization, the opal shows a reflectance peak centered at 616 nm with full-width-half-maximum (FWHM) of 39 nm, assigned to the stop-band of the photonic structure. Moreover, the spectrum background displays a Fabry–Perot pattern assigned to the interference occurring between light beams partially reflected from the top and bottom opal interfaces. This pattern can be used to estimate the opal thickness by applying the effective medium theory, which results in 4.5 μm.^10,57^ In analogy with the reflectance spectrum, the transmittance data display a minimum at 616 nm, which corresponds to the stop-band previously observed. The lack of fringes in the background of this spectrum is a common phenomenon that depends on the opal thickness. The spectrum is indeed dominated by light scattering, featuring an intensity reduction upon moving to the short wavelength side of the spectrum. The angular dispersion of the transmittance spectrum of the opal at increasing collection angle is reported in Fig. S4 and S5,† and further proves the presence of a stop-band. The hypsochromic dispersion of the stop-band is in full agreement with the literature.^35,58–60^Fig. 2b and c show two SEM micrographs of the synthetic opal where the face-centered-cubic lattice structure can be observed; such lattice arrangement is typical of both synthetic and natural opals based on silica or polystyrene nanospheres.^58^ Table S1† compare the calculated and experimental spectral position of the stop-band for opal fabricated by self-assembly of spheres having diameter 261 and 277 nm. Modifying the sphere diameter, the stop-band shift to the res part of the spectrum in agreement with calculated values.^10,59,61^ The experimental position of the stop-band is slightly blues-shifted with respect to the theoretical interval. This deviation can be ascribed to an underestimation of the effective refractive index of the spheres in the calculated values, where we did not consider the presence of RhDB. Indeed, the large refractive index of this molecule^62^ in the spectral region under investigation can increase the effective refractive index of the spheres,^63^ and in turn the spectral position of the stop-band making then the discrepancy between modelled and theoretical data reasonable.

**Fig. 3:**
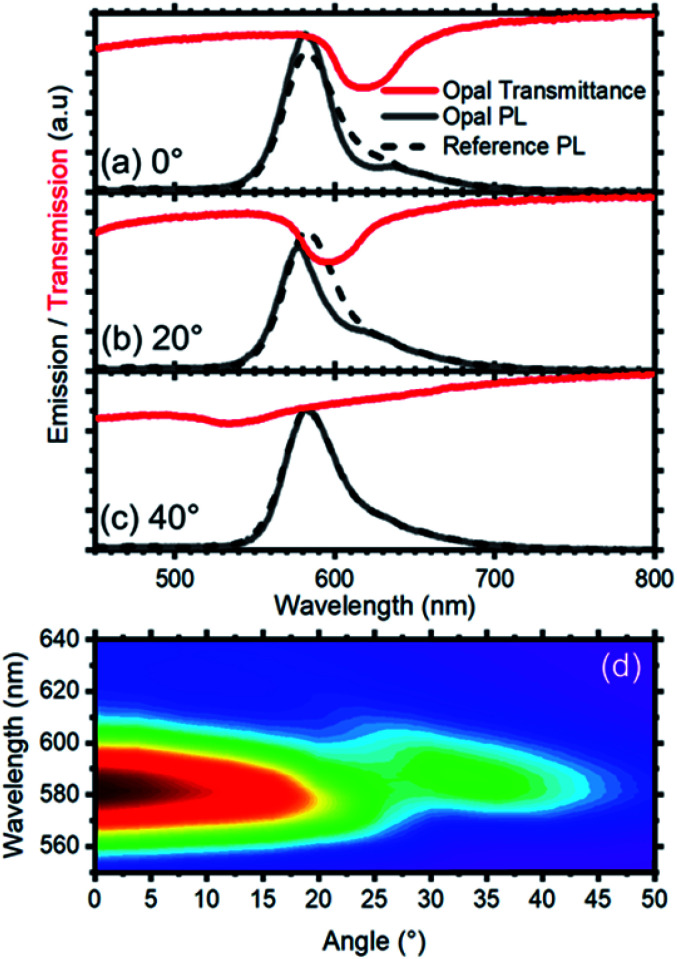
Photoluminescence spectra collected from the opal (full black curve) and the reference (dashed black curve) at different collection angles: (a) 0°, (b) 20° and (c) 40°. The respective transmission spectra are shown (red curves) to indicate the spectral position of the photonic band gap. (d) Photoluminescence intensity color map *vs.* collection angle where the dispersion of the photonic band gap induces an angle-dependent modification of the photoluminescence intensity and spectral shape.

The presence of a photonic stop-band spectrally superimposed to the photoemission of the RhDB embedded in the nanosphere shell modifies the line-shape of the latter. Fig. 3a compares the emission spectra of the opal (dashed line) with the one of not assembled spheres (continuous line). As mentioned above, the emission of not assembled spheres is peaked at 616 nm and possesses a FWHM of 40 nm. In contrast, the spectrum of the opal shows a suppression of the emitted intensity between 595 and 640 nm, and an enhancement in the spectral range between 595 and 550 nm (compare dashed and continuous lines in Fig. 1a). Both suppression and enhancement originate from the modified light–matter interaction occurring when the dye is within the photonic crystal. Indeed, in the frequency region close to the stop-band, the density of photonic states is thoroughly affected by the photonic structure.^41^ Within the stop-band, the density is dramatically reduced, also accompanied by a decrease of the photon emission, while at the stop-band edges the local density of photonic states is increased, with enhanced emission intensity. The overall effect can be thought as a squeezing of the photonic states from the stop band within the frequencies of its edges. Notice that in the opal the density of photonic states has different effects on the emission of the spheres depending on their position.^59,64–66^ The opal thickness has been estimated from the interference pattern reported in Fig. 3 and corresponds to 4.5 μm (21 lattice layers). Comparing this value with the Bragg length (*L*_B_), that is the attenuation of transmitted wave in a photonic crystal at the stop-band, it is possible to provide a qualitative estimation of the effects.^59^*L*_B_ can is calculated as:1
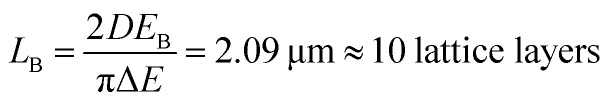
where *D* is the interplanar spacing, *E*_B_ the stop-band energy, and Δ*E* its full-width half-maximum of the stop-band. Because the opal consists in 21 lattice planes, this means that only the emission of the inner spheres is fully modified by the band gap, while the effect is lower as the sphere position approaches the surface. Since our set-up cannot disentangle such two signals, the measured emission is an average of the overall emission. These effects are related to the opal photonic band structure, which is angle dependent (photon momentum). Consequently, a substantial angular redistribution of the opal photoemission is expected.^41,58^ Indeed, the photoluminescence line shape modification follows the angular dispersion of the stop-band (Fig. 3b and c and ESI Fig. S4†). Hence, the suppression/enhancement effects can be spectrally tuned by increasing the collection angle of the emitted light. Fig. 3b reports the emission spectra collected at 20° for both opal (continuous line) and not assembled (dashed line) spheres. The emission spectrum of the opal is different from the one collected at normal incidence and reported in Fig. 3a. Indeed, the suppression shifts to the blue side of the spectrum with respect to the opal spectrum reported in Fig. 3a, as the stop-band also shifts with the angle of collection. As a consequence of the angular dispersion of the stop-band, at collection angle 40° or above, the band and the RhDB photoemission are not spectrally superimposed, and the emission of the latter becomes comparable to that of the not assembled spheres (Fig. 3c). Fig. 3d shows the full-angular dispersion of the opal photoemission as a contour plot where the wavelengths are reported as *y*-scale, while the collection angle as the *x* scale. The emission intensity is reported in false colors such that larger emission intensities are in red and green shades, while the smaller are in blue shades. In this plot the emission spans from 550 to 510 nm at normal incidence. Increasing the collection angle, the peak intensity decreases due to the suppression originated by the stop-band. At about 20°, the stop-band crosses the opal photoemission until 32° (see also ESI Fig. S5 and S6†). These findings agree with theory and previously published data.

**Fig. 4:**
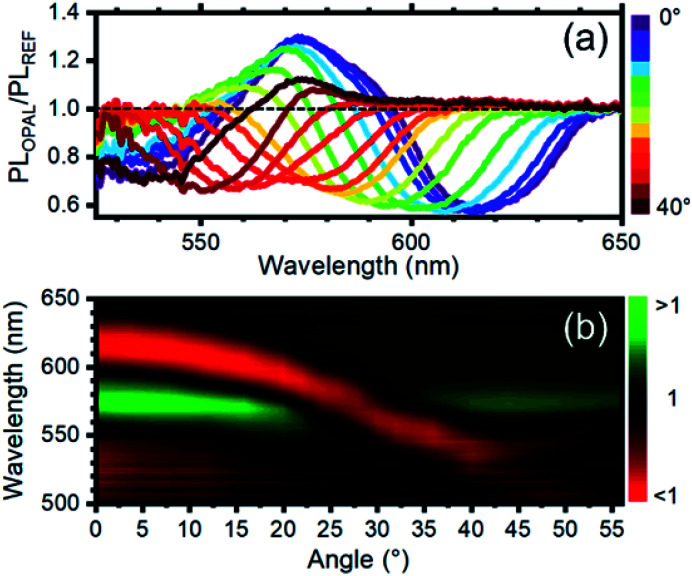
(a) Photoluminescence intensity modification *vs.* collection angle. A value >1 indicates photoluminescence enhancement, while a value lower <1 indicates photoluminescence suppression. The curves are obtained by diving the photoluminescence spectra of the opal and the reference after normalization at a collection of 60° where the photonic band gap does not affect the photoluminescence intensity and shape. (b) Photoluminescence enhancement/suppression map *vs.* angle. The observed photoluminescence suppression follows the angular dispersion of the photonic band gap (see ESI Fig. S4†).

To quantify the effect of the photonic structure on the RhDB emission and highlight emission and suppression effects, the PL ratio spectra, that corresponds to the ratio between the emission spectrum recorded at different angles divided by the reference spectrum,^58^ was calculated. At collection angle 0° (Fig. 4a) the fluorescence of the opal shows intensity enhancement up to 1.35 folds with respect to the reference between 595 and 540 nm. An intensity suppression of similar magnitude is observed between 595 and 640 nm, in agreement with Fig. 3. As previously observed, the maximum and minimum in these spectra, which correspond to emission and suppression, shift to the blue side of the visible spectrum increasing the collection angle. This behavior is clearly visible in the contour plot of Fig. 4b reporting the ratio spectra against the collection angle. Values smaller than 1 (emission suppression) are highlighted in red shades, while values larger than 1 (emission enhancement) appear in green shades. The suppression then moves from around 620 nm at normal incidence collection and reaches 520 nm at 56°. In turn, the enhancement also shifts accordingly.

To assess the possible effect of the opal on the radiative rate of the emitter, the emission decay of the structure was also measured as described in the Experimental section at the stop band wavelength and at its edges. Indeed, in agreement with theory, radiative rate enhancement implies both emission intensity enhancement and faster decay where the density of photonic states is larger. Accordingly, the emission decay should be slower where the density of photonic states is reduced.^41,67–70^ The decays collected at the two wavelengths are reported in Fig. S3,† and do not show substantial differences. Lack of radiative rate enhancement is indeed common in self-assembled structures and in photonic crystals with relatively low refractive index contrast.^69,71^

**Fig. 5:**
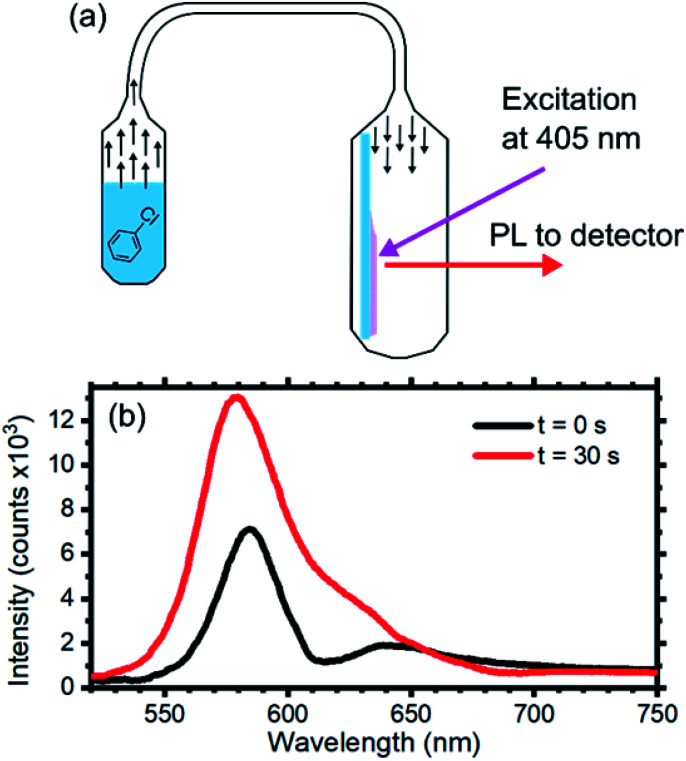
(a) Scheme of the chlorobenzene sensing test: the opal was inserted in a quartz cuvette directly connected to a vial containing the chlorobenzene. The opal was exposed to a constant solvent concentration of 53 mg l^−1^ over the test duration. (b) Photoluminescence spectra of the opal collected at 0° at increasing exposure times.

As mentioned in the introduction, as an example of future application of the core–shell system, we investigated the presence of an optical response of the opal upon exposure to chlorobenzene vapors.^72^ Several works have reported on the use opals for sensing of both liquid,^9,44^ and vapor analytes,^73–78^ thus making this field appealing for environment and anthropic systems safety. Our group already demonstrated that planar 1D polymer photonic crystals represents an efficient system to transduce the presence of chemical species to chromatic signal, also allowing label-free molecular recognition.^28–30,57,78–80^ The measurement set-up is schematized in Fig. 5a and consists of a sealed container, where the opal is placed, connected to a reservoir of the liquid analyte that allows its vapors to reach to sample in the second chamber. Connecting the vapor reservoir to the sample chamber, chlorobenzene vapor diffuses towards the opal. Once the vapor reaches the sample chamber, it infiltrates within the opal, modifying its effective refractive index, thus its optical response and photoluminescence spectrum.

Fig. 5b displays the opal emission spectra collected before (black line) and after (red line) 30 s of exposure to chlorobenzene vapor with a concentration of 53 mg ml^−1^. The initial spectrum is consistent with the data previously discussed. After 30 s of exposure the signature of the emission suppression assigned to the stop band detectable at about 610 nm fades and the overall emission intensity increases. The first effect can be ascribed to capillary condensation within the interstices between the sphere. This effect reduces the dielectric contrast of the opaline structure generating a homogenous effective medium.^25^ Capillary condensation has been already demonstrated to be an efficient sensing mechanism to alcohols for opals.^42^ The second effect can instead be attributed to better light extraction. Indeed, reducing the dielectric contrast at the sphere interfaces favors photon escaping in the entire system.^81^

The responsivity of the opal to chlorobenzene vapor is very promising for the study and development of opaline photonic structure for sensing of vapor pollutants based on fluorescence variations.

## Conclusions

We have demonstrated the engineering of nanospheres containing a shell functionalized with rhodamine B. The low polydispersity of the sphere diameter allows self-assembling of fluorescent artificial opals with peculiar properties. Indeed, by appropriately selecting the sphere diameter we obtained a spectral superimposition of the dye photoemission spectrum with the opal stop-band. This superimposition leads to a spectral and angular redistribution of RhDB emission line-shape and intensity, both tunable exploiting the angular dispersion of the photonic band structure. Moreover, such opals show sensitivity to chlorobenzene vapor resulting in a reduction of the dielectric contrast of the opal thus providing a restoration of the original dye emission line-shape and intensity. These results are promising for the development of solution-processed photonic sensors for vapor pollutants.

## Conflicts of interest

There are no conflicts to declare.

## Supplementary Material

RA-010-D0RA02245D-s001
