# Supplementary material for: Core–shell silica–rhodamine B nanosphere for synthetic opals: from fluorescence spectral redistribution to sensing
Source: RSC Adv. 2020 Apr 16;10(25):14958–64. doi: 10.1039/d0ra02245d (PMC9052040; doi:10.1039/d0ra02245d)
Supplement: RA-010-D0RA02245D-s001 [file RA-010-D0RA02245D-s001.pdf]

Electronic Supplementary Material (ESI) for RSC Advances.
This journal is © The Royal Society of Chemistry 2020

Core-shell silica-rhodamine B nanosphere for synthetic opals: from fluorescence spectral redistribution to sensing

P. Lova[a), #], S. Congiu[a)], K. Sparnacci[b), #], A. Angelini[c)], L. Boarino[c)], M. Laus[b)], F. Di Stasio[a),*], D. Comoretto[a)]

a) Dipartimento di Chimica e Chimica Industriale, Università di Genova, via Dodecaneso 31, 16146 Genova

b) Dipartimento di Scienze e Innovazione Tecnologica, Università del Piemonte Orientale, viale T. Michel 11, 15121 Alessandria, Italy, INSTM UdR Alessandria

c) Nanofacility Piemonte, Istituto Nazionale Ricerca Metrologica, Strada delle Cacce 91, 10135 Torino

[#] corresponding author
* Present Address: Nanochemistry Department, Italian Institute of Technology, Genova (I)

Electronic Supporting Information

Supporting Information Figure S1 shows scheme of the multistep synthetic procedure employed to obtain the fluorescent silica nanospheres.

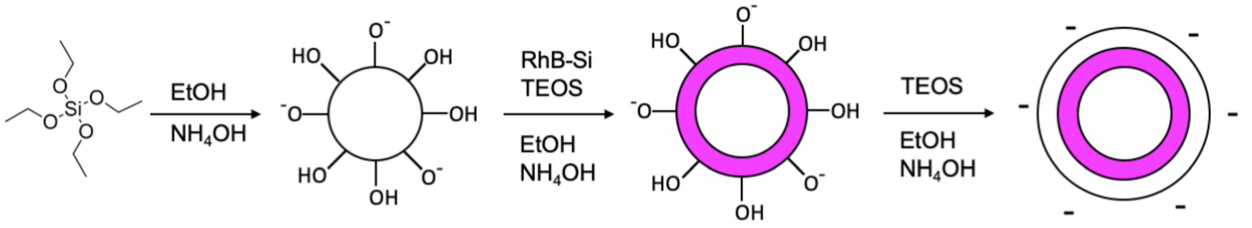

Figure S1: Schematic for the multistep synthetic procedure for the fluorescent silica nanospheres

Synthesis of the fluorescent silica nanoparticles

Materials: All reagents and solvents were purchased from Sigma-Aldrich and used as received. MilliQ water (18 MΩcm^{-1}, Barnstead) was thoroughly used. Glassware was thoroughly cleaned with concentrated nitric acid, then with 1 M hydrofluoric acid, rinsed with MilliQ water and oven-dried.

***Synthesis of Silica Core*:** 32.5 ml of water and 8.3 ml of ammonium hydroxide (16.3 M NH_3, checked by potentiometric titration) were mixed and absolute ethanol was added to a volume of 320 ml. The solution was introduced in a 500 ml round-bottom flask and heated to 30.0°C in an oil bath with a stirring rate of 200 rpm, then 20.0 ml of TEOS was added and the reaction was carried out for 6 h.

Synthesis of the Fluorescent Silica Shell: The functional fluorescent dye (RhBSi) was prepared dissolving 10.6 mg of rhodamine B isothiocyanate ($2\cdot10^{-5}$ mol) and 350 μL of APTMS (2.0 mmol) in 6 ml absolute ethanol. The solution was placed in a 10 mL screw-cap vial and the reaction was performed at room temperature in the dark under stirring for 3 h.

This solution was then added, together with 1.0 ml of TEOS to the flask contining the silica core particles, and the reaction was let to proceed for further 16 h. At the end of the reaction the suspension was purified by centrifugation and redispersion in ethanol until no rhodamine B could be detected in the supernatant.

Growth of the External Silica Layer: To an ethanolic suspension containing 2.0 g of the fluorescent silica nanoparticles 8.3 ml of ammonium hydroxide and 32.5 ml of water, then absolute ethanol was added to a volume of 320 ml. were added and the suspension heated to 30°C. The solution was introduced in a 500 ml round-bottom flask and heated to 30.0°C under stirring, then 1.5 ml of TEOS was added. The reaction was carried out for 6 h then the suspension was purified by centrifugation and redispersion in ethanol for three times.

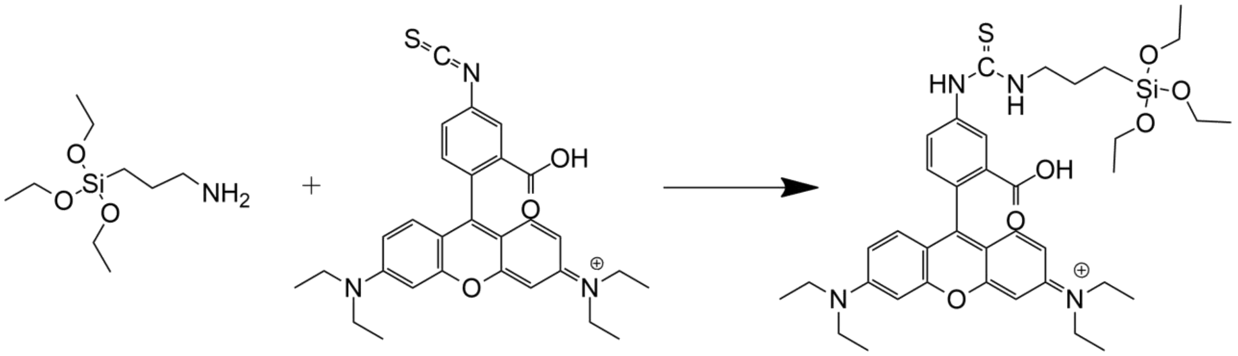

Figure S2: Synthesis of functional fluorescent dye (RhBSi)

Supporting Information Figure S3a shows the PL spectrum of the opal and of the nanosphere suspension. The effect of the stop band is observed as a minimum in the PL spectrum of the opal. Fig. S3b and S3c show the PL decay traces as recorded at 588 nm (PL peak) and 626 nm (stop band)

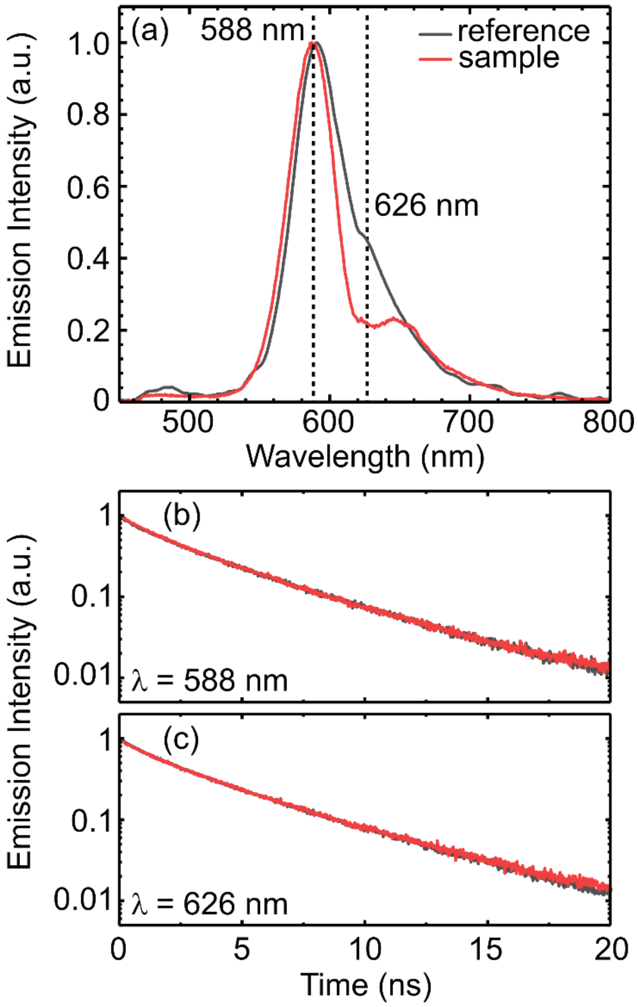

Figure S3: (a) comparison between the opal PL structure (red line) and the emission of the non-assembled microspheres (black line). Photoemission decay for the collected at 588 nm (b) and at 626 nm (c). The two wavelengths correspond to the stop band edge and to the center of the latter, respectively.

Table S1 reports the experimental and calculated PBG spectral positions for opals made with microspheres with diameter of 261 and 271 nm.[1-3] The calculated data were retrieved for spheres entirely made of silica. The relatively large polydispersity of the sphere diameters in the two samples (ca 5-6 %) is reflected on the spectral positions of the PBGs, that present standard deviation up to 13 nm for the experimental data and up to 34 nm for the calculated ones. This effect can be described to the synthetic routes employed for the core-shell spheres, indeed synthesis of bare silica particles commonly provided polydispersity well below 5%.[4] The experimental position of the PBG is slightly blues-shifted with respect to the theoretical interval. This deviation can be ascribed to an underestimation of the effective refractive index of the spheres in the calculated values, where we did not consider the presence of RhDB. Indeed, the large refractive index of highly polarizable RhDB [5] in the spectral region under investigation can increase the effective refractive index of the spheres,[6] and in turn the spectral position of the PBG making then the discrepancy between modelled and theoretical data reasonable.

Table S1: Experimental and calculated spectral position for opal made with spheres with different diameter

Sphere diameter (nm)	λ_{PBG} (nm)	Calculated λ_{PBG} (nm)
277 ± 13	630 ± 13	610 ± 33
261 ± 15	613 ± 3	575 ± 34

Supporting Information Figure S4 shows the opal transmittance spectra as a function of the incidence angle for unpolarized light.

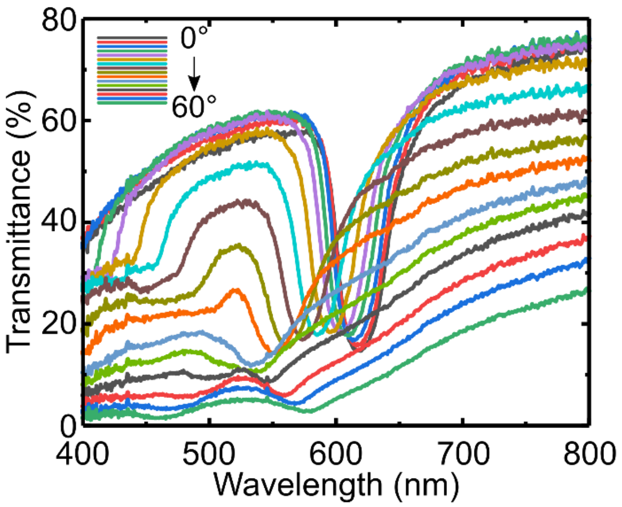

Figure S4: Angular resolved transmittance spectra of the opal: spectral dispersion of the opal stop band as a function of the incidence angle for unpolarized light.

Supporting Information Figure S5 shows the polarized angle dependence of the opal transmittance spectra.

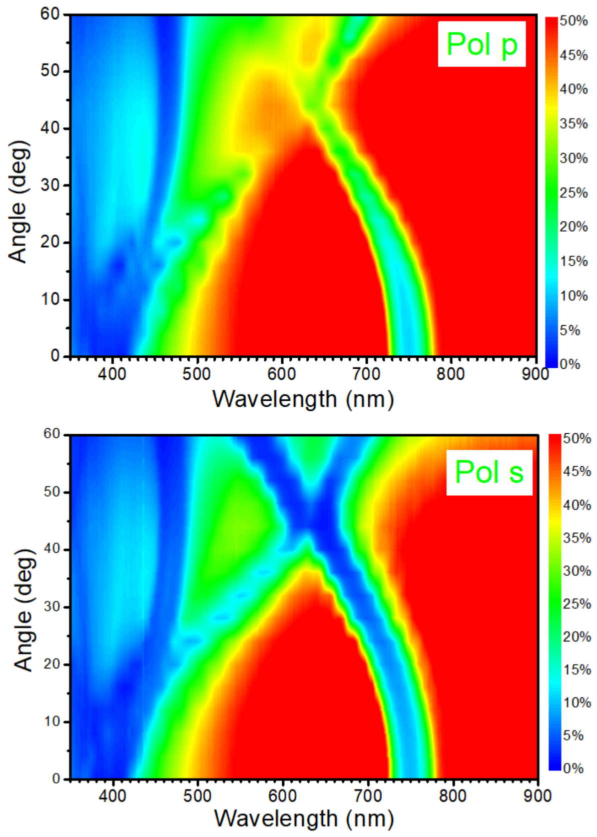

Figure S5: Angular resolved transmittance spectra of the opal: contour plots for p-polarized light (top panel) and s-polarized light (bottom panel).

Supporting Information Figure S6 shows the opal PL spectra as a function of the detection angle.

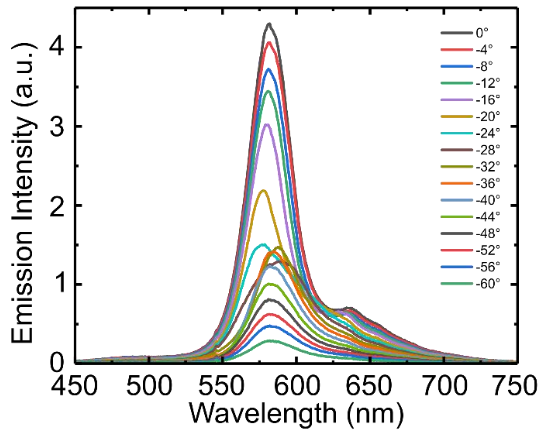

Figure S6: Angular PL spectra of the opal structure

REFERENCES

1. E. Pavarini, L. C. Andreani, C. Soci, M. Galli, F. Marabelli and D. Comoretto, *Physical Review B*, 2005, **72**.
2. D. Comoretto, ed., *Organic and Hybrid Photonic Crystals*, Springer International Publishing, Cham, Switzerland, 2015.
3. V. Morandi, F. Marabelli, V. Amendola, M. Meneghetti and D. Comoretto, *J. Phys. Chem. C*, 2008, **112**, 6293-6298.
4. K. Sparnacci and M. Laus, in *Organic and Hybrid Photonic Crystals*, ed. D. Comoretto, Springer, Cham, 2015, ch. 5, pp. 103-125.
5. A. A. M. Farag and I. S. Yahia, *Optics Communications*, 2010, **283**, 4310-4317.
6. R. J. Gher and R. W. Boyd, *Chem. Mater.*, 1996, **8**, 1807–1819.
